# Body Composition and Body Weight Changes at Different Altitude Levels: A Systematic Review and Meta-Analysis

**DOI:** 10.3389/fphys.2019.00430

**Published:** 2019-04-16

**Authors:** Tobias Dünnwald, Hannes Gatterer, Martin Faulhaber, Marjan Arvandi, Wolfgang Schobersberger

**Affiliations:** ^1^Institute for Sports Medicine, Alpine Medicine & Health Tourism, UMIT – University for Health Sciences, Medical Informatics and Technology, Hall in Tirol, Austria; ^2^Institute of Mountain Emergency Medicine, EURAC Research, Bolzano, Italy; ^3^Department of Sport Science, University of Innsbruck, Innsbruck, Austria; ^4^Institute of Public Health, Medical Decision Making and HTA, Department for Public Health, Medical Decision Making and Health Technology Assessment, UMIT – University for Health Sciences, Medical Informatics and Technology, Hall in Tirol, Austria; ^5^Tirol Kliniken GmbH Innsbruck, Innsbruck, Austria

**Keywords:** weight loss, body composition, high altitude, nutrition, hypoxia, exercise

## Abstract

Changes in body composition and weight loss frequently occur when humans are exposed to hypoxic environments. The mechanisms thought to be responsible for these changes are increased energy expenditure resulting from increased basal metabolic rate and/or high levels of physical activity, inadequate energy intake, fluid loss as well as gastrointestinal malabsorption. The severity of hypoxia, the duration of exposure as well as the level of physical activity also seem to play crucial roles in the final outcome. On one hand, excessive weight loss in mountaineers exercising at high altitudes may affect performance and climbing success. On the other, hypoxic conditioning is presumed to have an important therapeutic potential in weight management programs in overweight/obese people, especially in combination with exercise. In this regard, it is important to define the hypoxia effect on both body composition and weight change. The purpose of this study is to define, through the use of meta-analysis, the extent of bodyweight -and body composition changes within the three internationally classified altitude levels (moderate altitude: 1500–3500 m; high altitude: 3500–5300 m; extreme altitude: >5300 m), with emphasis on physical activity, nutrition, duration of stay and type of exposure.

## Introduction

Short- as well as long-term exposure to hypoxic environments cause comprehensive physiological alterations (Swenson and Bärtsch, 2014). Both normobaric (i.e., simulated altitude) and hypobaric (i.e., real and simulated altitude) hypoxia can lead to a lower partial pressure of oxygen in blood and tissues. As an acute compensatory response, ventilation increases and sympathetic activation causes an altitude dependent increase in cardiac output in lowlanders exposed to high altitude (Baggish and Wolfel, 2014). Hyperventilation is one of the most important factors for ensuring oxygen supply to the tissue. Peripheral chemoreceptors, located in the carotid bodies, respond to reductions in arterial partial pressure of oxygen. When a fall in SaO_2_ is detected, signals stimulate ventilation and lead to sympathetic activation (Kara et al., 2003), hence increasing metabolic demands. In dry and cold environments, the increased ventilation may be accompanied by increased loss of water (i.e., insensible water loss) (Kayser, 1994). These ventilatory and cardiovascular responses ensure that metabolic demands of organs and tissues are met at rest and during exercise at acute altitudes. During sustained hypoxia, cardiac output decreases to levels approaching normoxia. This adaptive response is facilitated by stimulated erythropoiesis, increases in red cell mass as well as by further increases in ventilatory response to hypoxia (Baggish and Wolfel, 2014). In addition to these adaptations, hypoxic environments are frequently shown to influence a person’s body composition (e.g., reductions in body weight, fat free mass (FFM), fat mass (FM), muscle mass (MM) and/or body water) (Hamad and Travis, 2006).

**FIGURE 1:**
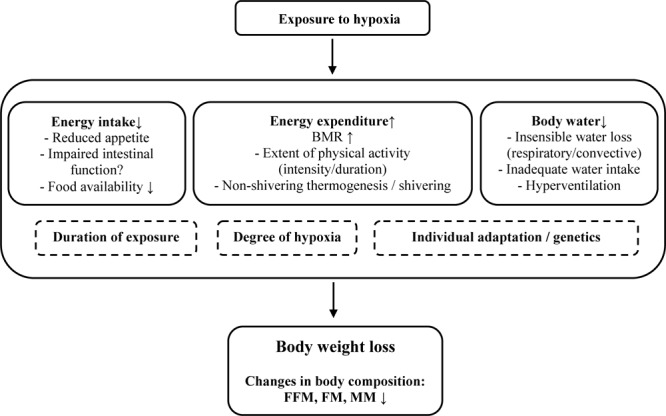
Potential factors being involved in bodyweight and body-composition changes at altitude. BMR, basal metabolic rate; FFM, free fat mass; FM, fat mass; MM, muscle mass.

Proposed factors responsible for these changes in body composition vary but mainly include an increased basal metabolic rate (BMR) and a negative energy balance (i.e., mismatch of energy intake and energy expenditure) (Kayser and Verges, 2013) (Figure 1). Loss in body water may arise from inadequate water intake, hyperventilation or insensible water loss (respiratory and surface water loss) (Kayser, 1994; Gatterer et al., 2013). A decrease in plasma volume is supposed to arise from reduced activity of aldosterone and increased atrial natriuretic peptide concentration, leading to enhanced fractional Na-excretion and “high altitude diuresis” (Siebenmann et al., 2017). However, water retention at altitude may also contribute to hyperhydration and weight gain, at least during the first days at altitude (Gatterer et al., 2013). The increased BMR is most pronounced during acute hypoxia (Brooks, 2014) and less so during prolonged exposure (Hannon and Sudman, 1973; Butterfield et al., 1992). A higher initial BMR has been related to increased sympathetic activity with higher catecholamine levels during the first days of exposure (Mawson et al., 2000; Kayser and Verges, 2013), that is influenced by the degree of hypoxia (Butterfield et al., 1992). Sympathetic activity is attenuated during longer exposures to high altitude, resulting in diminished BMR. Acute increases in BMR might also be explained by the stress of an initial energy deficit, whereas the decline in BMR during prolonged stays could depend, at least in part, on personal fitness, such that those with a higher fitness level will experience larger reductions in direction to baseline levels (Mawson et al., 2000). In addition to an increased BMR, at least at altitudes above 5000 m, a negative energy balance may result from a reduced energy intake due to either a reduced appetite (Westerterp-Plantenga et al., 1999) or, in part, by impaired intestinal function (Hamad and Travis, 2006). In order to negate loss of heat and maintain body temperature in cold environments, an additional increase in energy expenditure may arise due to involuntary shivering (non-shivering thermogenesis), which is activated to increase heat production and involves depletion of fat storages (Burtscher et al., 2018). Moreover, increased energy expenditure during extensive high-altitude hiking could be responsible for a negative energy balance leading to changes in body composition (Westerterp et al., 1992; Hamad and Travis, 2006; Kayser and Verges, 2013).

It follows then that, in addition to energy balance and activity level, the degree of hypoxia seems to play an obvious role in body composition changes at different altitudes. For instance, reports indicate that body weight loss is a function of the absolute altitude level, and further depends on the duration of the stay, individual adaptation to hypoxia, daily variations in body mass, hydration and type of exposure (Kayser, 1994). Additionally, muscle wasting seems to ensue predominantly at altitudes exceeding 5000 m, whereas catabolic mechanisms may be of minor importance at moderate altitudes with exposure to lower levels of hypoxia (D’Hulst and Deldicque, 2017). Muscle catabolism, which may lead to muscle wasting, can be observed during adaptation to hypoxic exposures when a focus is on restricting the effect of decreased oxygen delivery (Favier et al., 2015). Reductions in muscle mass may lessen BMR (Rolfe and Brown, 1997) and, in addition, are purported to lead to a reduced oxygen diffusion distance that corrects the effect of a decreased oxygen supply to the muscle fibers, both of which illustrates a protective mechanism for ensuring oxygen availability. Muscle catabolism may arise due to a negative energy balance resulting from increased energy expenditure, high levels of physical activity and inadequate nutritional intake. After glycogen and fat stores are depleted, protein stores will be catabolized in order to cover energy expenditure (Ocobock, 2017). In addition, inhibition of skeletal muscle protein synthesis and a preference to use muscle mass as an energy source (rather than fat mass) during hypoxia are both potential factors in exacerbating muscle wasting (Murray, 2009).

Despite such findings, the effect of the altitude level (AL) on body composition has not yet been well reviewed. Therefore, this study aims to provide an overview of the influence of AL on body composition changes. Obviously, it is not possible to completely isolate the influence that altitude alone has on changes in body composition because most studies describe a number of influencing factors. In fact, according to literature, changes in body composition are the culmination of several single contributors (Hamad and Travis, 2006), of which the degree of hypoxia may be one of the most relevant. Therefore, our systematic review takes a meta-analytic and meta-regression approach to focus on body compositional changes as a function of different AL’s (i.e., moderate altitude (1500–3500 m) high altitude (3500–5300 m) and extreme altitude (>5300 m), as well as taking into account other factors such as duration of stay, active or passive exposure and nutrition.

## Methods – Systematic Review

A systematic literature search was performed in PubMed, The Cochrane Library (central register of controlled trials), Web of Science (Core collection) and EMBASE, and included studies published prior to January 2019. As the present review focuses on altitude dependent variations in body composition, selected abstracts, titles and full texts had to concur with the following inclusion criteria: original research article, available full text, human subjects, English language, hypobaric (simulated altitude or altitude exposure) or normobaric hypoxia, active (exercise) and passive hypoxia, indication of AL, and assessment of at least one of the following parameters: FFM, FM, MM, BMI, body weight or assessment of metabolic response. Exclusion criteria included the absence of information on body composition and/or body weight, studies involving neonates, newborns, high altitude natives or animals, permanent sojourns at altitude and/or disease related hypoxia. The systematic literature search was based on the following search strategy: controlled vocabulary (i.e., MeSH and EMTREE Terms) as well as specific text words (including hypoxia, “altitude training,” altitude, “body composition,” “fat mass,” “body mass,” “body weight,” “metabolic response,” “physiological response,” exercise, healthy, overweight, obesity, athlete, patient) were included and systematically combined by the use of Boolean operators (AND/OR).

## Study Selection

**FIGURE 2:**
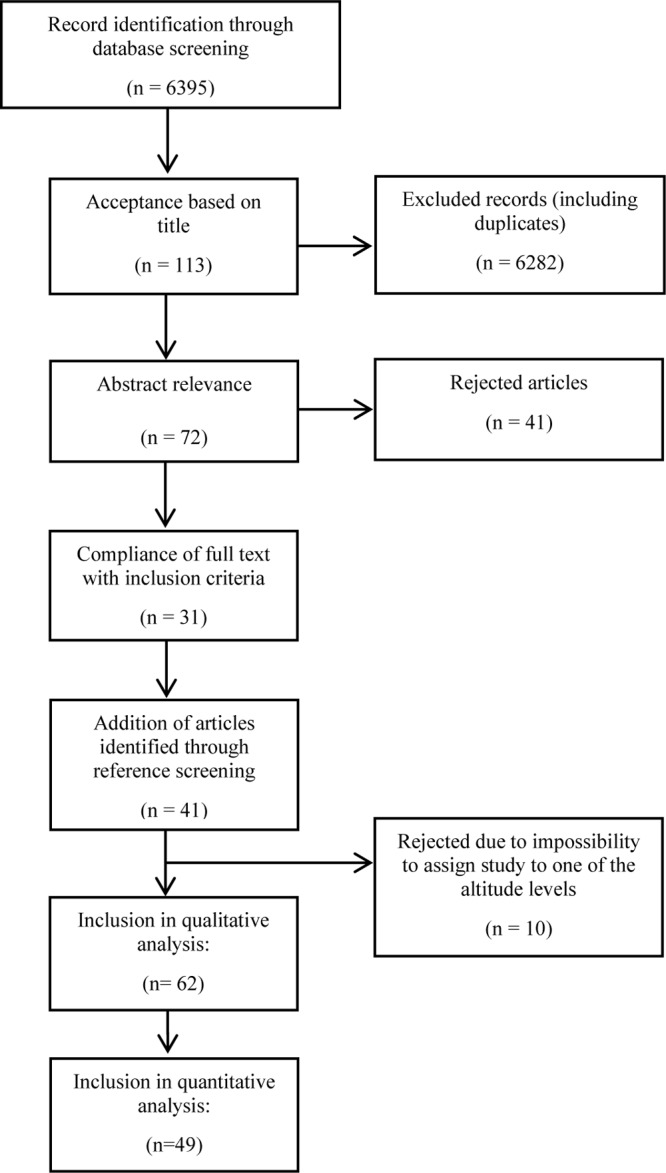
Flow chart representation of literature search.

In total, 6395 studies were found in the initial search and screened for relevant titles by two researchers (HG, MF) independently. After excluding 6282 articles by title (including duplicates), the abstracts of the remaining 113 studies were screened for relevance. Thereof, 31 fulfilled the inclusion criteria. Articles were removed due to the following criteria: animal studies, studies involving infants, patients with COPD or high altitude natives, content was not relevant for the review, commentaries or reviews, studies involving (endurance) athletes and/or studies implementing short-term (intermittent) exposures <12 h/day. Complementary to the data base searches, reference lists of selected review articles (Hamad and Travis, 2006; Westerterp and Kayser, 2006; Hill et al., 2011; Kayser and Verges, 2013; Heinonen et al., 2016; D’Hulst and Deldicque, 2017; Hobbins et al., 2017; Pasiakos et al., 2017) were carefully screened and relevant original articles were included that complied with the determined inclusion criteria (*n* = 25). In addition, another 16 full texts were included by reference list searching of included studies. Articles where it was not possible to assign changes in bodyweight and body-composition to a corresponding altitude level were also excluded (*N* = 10). A flow chart of the search is illustrated in Figure 2. Most of the selected articles involved an uncontrolled before-and-after study design. Overall, 62 studies were included in the qualitative analysis, and 49 were included in the quantitative analysis a s not all studies provided sufficient information needed for a meta-analysis (Tables 1A–C).

**Table 1A:** Changes in body weight and body composition during moderate altitude.

Authors	Participants M/F, Age [years]	Type of exposure		Diet manipulation	Altitude level [m]	Duration [days]	Body weight [Δkg (Δ% of BW)]	Body composition [Δkg (Δ%)]			
		Passive	Active	Yes/No				FFM	FM	MM	TBW
**Moderate altitude**											
Ocobock (2017)	18-31 yrs G1(temperate): 37 M G2(cold): 22 M G3(temperate): 16 F G4 (cold): 6 F		Endurance Temperate Cold Temperate Cold	No (recordings)	3205-3658	84; G1: 112; G2: 84; G3: 112; G4:	–2.8 (–3.6) –2.3 (–3.0) –1.1 (–1.6) –2.1 (–3.0)		–1.9 (–21.0) –0.8 (–9.6) –1.6 (–9.5) –2.6 (–14.6)	–0.9 (–1.4) –1.5 (–2.3) 0.5 (1.0) ns 0.6 (+1.2)	
Jacobs et al. (2016)	8 M, 1 F 26.9 ± 3.7 yrs		Sea level activity	No	3454	28	↔	↔	↔		
Mekjavic et al. (2016)	11 M 23.7 ± 4.0 yrs	Stay at simulated altitude		No (recordings, *ad libitum*)	2800 3000 3200 3400	(10) 2 2 3 3	–0.9 (–1.2) ns	–0.8 (–1.3) ns	–0.1 (–0.9) ns		
Kasprzak et al. (2015)	9 M 28.0 ± 4.3 yrs		Ascents	No (dietary recordings, *ad libitum*)	3200 up to 3800	14	–1.6 (–2.2)	–1.1 (–1.9) ns	–0.5 (–4.0) ns		–0.7 (–1.5) ns
Chia et al. (2013)	10 M 14.9 ± 0.4 yrs		Daily Swimming	No	2300	21	0.1 (0.1) ns	0.8 (+1.5)	–1.7 (–11.4)		
Chen et al. (2010)	12 M 31 ± 1.1 yrs		Daily hiking	No (*ad libitum*)	between 2200 and 3800	25	–1.4 (–2.0) ns		–2.1 (–15.3)		
Lippl et al. (2010)	20 M 55.7 ± 4 yrs	Stay at natural altitude		No (no diet restrictions)	2650	7	–1.5 (–1.4)		na (–0.3) ns	na (0.1) ns	
Greie et al. (2006)	36 M 56 yrs (36-66 yrs)		12 hiking sessions	No (standardized nutrition menues, no dietary restrictions)	1700+	21	–3.3 (∼-3.3)				
Gunga et al. (2003)	22 M 54 yrs (40-64 yrs)		Hiking and swimming	No (*ad libitum*)	∼1700	21	–0.4 (–0.4)	1.6 (2.4) ns			0.6 (1.2)
Tanner and Stager (1998)	5 M 40.0 ± 5.5 yrs		Trekking	No (Dietary recordings, no attempt to manipulate diet, goal: 5500 kcal/day)	2200-4300	21	–4.2 (–5.4)	–3.1 (–4.7)	–1.0 (–8.2)		
Hoyt et al. (1994)	6 M 31 ± 4 yrs		Strenuous winter exercise	Yes (prescribed diet, US military field ration (22.7 MJ/d) dietary records)	2500-3100	5	–1.8 (–2.3)	–0.4 (–0.6) ns	–1.4 (na)		–0.1 (–0.2) ns
Schena et al. (1992)	11 M, 5 F 35.8 ± 5.6 yrs G1: dietary supplementation G2 placebo		Trekking	Yes (dietary recordings, amino acid supplementation)	2500-4000 (4 d < 3000)	21; G1 21; G2	–1.0 (–1.6) –1.8 (–2.8)	0.7 (1.5) ns –0.2 (–0.4) ns	–1.8 (–11.8) –1.6 (–10.2)		
Greenleaf et al. (1978)	6 M 21-23 yrs G1: *n* = 3 G2: *n* = 3		Running (2 × 30 min/day) simulated altitude	Yes controlled diet (3309 kcal/day)G1 with fluid replacement G2 drank *ad libitum*	2287	8	–0.6 (–0.9)				

*Studies presented in square brackets are not included in the meta-analysis due to missing data. M, male; F, female; yrs, years (mean ± SD or min-max) d, days; ns, non-significant; n.a., no data available; Δkg, mean changes in BW, FFM, FM, MM and TBW before and after data collection or mean changes over a defined period; Δ%, mean change in percent compared to baseline or to a defined period; BW, bodyweight; FFM, fat free mass; FM, fat mass; MM, muscle mass; TBW, total body water; G, study groups. If no data were available but direction of change was illustrated, arrow is indicated: ↔ no change.*

**Table 1B:** Changes in body weight and body composition during high altitude.

Authors	Participants M/F, Age [years]	Type of exposure		Diet manipulation	Altitude level [m]	Duration [days/wk]	Body weight [Δkg (Δ% of BW)]	Body composition [Δkg (Δ%)]			
		Passive	Active	Yes/No				FFM	FM	MM	TBW
**High altitude**											
Beidleman et al. (2017)	12 M, 5 F 22.9 ± 1.5 yrs		Several 2-3 mile hikes above 3500 m	No (*ad libitum*)	4300	12	–3.0 (-4.0)				
Berryman et al. (2017)	G1(standard protein): 8 M 23.1 ± 3.3 yrs G2 (higher protein diet): 9 M 23.7 ± 7.3 yrs		low-moderate	Yes (both groups were subjected to a 40% energy deficit)	4300	21; G1 21; G2	–7.2 (-8.3) –7.1 (-8.9)	–4.0 (-6.3) –3.3 (-5.6)	–3.3 (-16.5) –3.9 (-22.5)		
Tam et al. (2016)	7 F 36.3 ± 7.1 yrs		Moderate intensity	No	598 4132	12 14	–0.6 (-0.9) ns 1.2 (-1.9) ns	–1.9 (-4.0) ns +0.5 (1.1) ns			
Debevec et al. (2014a)	11 M 27 ± 6 yrs	a): bed rest c):bed rest (simulated altitude)	b): low-moderate (simulated altitude)	Yes (individ. tailored, strictly controlled, standardized diet)	3 different conditions 1: normoxia 2: 4300 3: 4300	21; a) 21; b) 21; c)	na (-3) na (-4) na (-5)	na (-4) na (-5) na (-5)	na (↔) na (↔)		
Debevec et al. (2014b)	11 M 24 ± 2 yrs a) b) c)	a):bed rest c).:bed rest	b):low-mod.	Yes (individually tailored, controlled, standardized diet)	3 different conditions a) normoxia b) 4000 c) 4000	10; a): 10; b) 10; c)	–1.4 (-2.0) –1.5 (-2.1) –2.0 (-2.8)	–2.4 (-4.3) –2.1 (-3.8) –2.1 (-3.8)			
Subudhi et al. (2014)	16 M 20.8 ± 1.4		Physically active	No	5260	16 d	–2.4 (-3.4)	–2.1 (-4.9)	–1.4 (-15.0)		
Wing-Gaia et al. (2014)	10 M, 8 F 44.5 ± 10.4 yrs (G1+G2) G1: *N* = 10, (leucine supplementation) G2: *N* = 8, (control)		Trekking	Yes (dietary recording, leucine supplementation)	4140 (2835-5364) 1 d < 3000	13; G(1+2): 13; G1: 13; G2:	–1.9 (-2.2) na (-2.2) na (-2.3)	–1.0 (-1.7) na (-1.2) na (-2.1)	–0.8 (-4.0) na (-5.4) na (-2.9)		
Boos et al. (2014)	33 M, 14 F 34.5 ± 9.3 yrs		Graduated ascent, start at 3600 m	No	3833 4450 5129	10	0.5 (0.7) 0.1 (0.1) 0.9 (1.2)				
[De Mol et al. (2012)]	8 M, 5 F 48.2 ± 9.7 yrs		Trekking expedition	No	4167	12	(na) ↔				
Levett et al. (2012)^∗^	G1: staff, 3 M, 3 F 39.5 ± 12.4 (G2: climber) 11 M, 1 F 38.0 ± 4.9		Ascent/stay ascents	No	1300-5300 up to 6400-8848	G1: 13 ascent/6 stay G2: 66	–0.9 (-1.2) ns –9.6 (-11.7)^#^				
Ermolao et al. (2011)	12 F 21. 5 ± 3.1 yrs		5 d ascent+ aerobic/strength moderate/intense exercise	No	5050	21	–1.0 (-1.6) ns	–0.4 (-0.9) ns	–0.7 (-4.1) ns	–0.2 (-1.3) ns	
Smith et al. (2011)	7 M, 3 F 37 ± 9 yrs (26-49 yrs)		Expedition	No	353-4000 4000-4750 4750-5300	after 3 d after 6 d after 9 d	–1.4 (-2.0) ns –1.4 (-2.0) –3.1 (-4.5)				
Ge et al. (2010)	160 M 32 ± 6 yrs G1: *N* = 85 (sea level residents) G2: *N* = 35 moderate altitude (altitude residents)		Construction work 5 h/d	No	4678	33; G1: 33; G2:	–7.1 (-10.6) –1.4 (-2.2)				
Holm et al. (2010)	9 M 24-30 yrs	Stay at natural altitude		Yes (registered food intake, matched amount of food as during sea level, similar diet as at sea level)	4559	7-9	–0.4 (-0.5) ns	–0.1 (-0.2) ns	–0.2 (-1.5) ns		
Mizuno et al. (2008)^∗^	15 M 25-45 yrs G1: *N* = 7 G2: *N* = 8		G1:little walking (base camp) G2:climbing/ ascents	No	5250 5250 up to 6400-7500^#^	75; G1: 75; G2:	–7.7 (-10.1) –5.4 (-7.1)				
Vats et al. (2007)	G1:placebo 10 M 23.8 ± 3.6 yrs G2:supplement (NAC) 10 M 22.5 ± 1.9 yrs G3:supplement (Vit E) 10 M 22.4 ± 1.9 yrs		Stay 3600 (+exercise), ascend to 4580	Yes (N-acetyl cysteine/vitamin E supplementation)	HA:3600 HA:4580 HA:3600 HA:4580 HA:3600 HA:4580	7, G1: 2, G1: 7, G2: 2, G2 7, G3: 2, G3:	–2.7 (-4.4) –3.1 (-5.0) –1.8 (-2.8) –2.3 (-3.6) –1.6 (-2.6) –2.1 (-3.4)				
Barnholt et al. (2006)	(adequately fed) 7 M 21.3 ± 2.8 yrs	Stay at natural altitude		Yes (prescribed, increased caloric intake)	4300	21 baseline - d 21	–0.8 (-1.1) ns	–1.4 (-2.1)	0.5 (7.4) ns		
Fulco et al. (2005)	16 M G1 (*n* = 8): 25.1 ± 6 yrs G2 (*n* = 8): 25.3 ± 6 yrs		Increased energy expenditure by 30-40% (∼ 4,567 kcal/day, e.g., running, hiking	Yes, standardized, diet individualized amounts (but voluntary reduced by ∼750 kcal/day versus sea level)	4300	10	–2.3 (-3.0)				
Shukla et al. (2005)	30 M 23.3 ± 2.5 yrs	Stay at natural altitude		No	3600 4300	2 7	–2.12 (-3.3)				
Lundby et al. (2004)	6 M, 2 F 25 ± 8 yrs		sea level activity	No	4100	56	–1.0 (-1.3) ns				
Fulco et al. (2002)	G1: 10 M 22.6 ± 4 yrs G2: 7 M 21.1 ± 3 yrs		Program of strenuous exercise (cycle ergometry, treadmill walking, and weight lifting)	Yes, G1: energy deficit by ∼1500 kcal/day G2: adequate kcal/day to maintain BW	4300	21; G1: 21; G2:	–6.6 (-8.2) –1.0 (-1.3) ns	–4.6 (-6.5) –0.8 (-1.2) ns			
Mawson et al. (2000)	16 F 21.7 ± 0.5 yrs		Treadmill, ergometer, strength training	Yes (controlled diet, specified quantities of standardized diet; increased energy intake)	4300	12	0.41 (0.7) ns				
Westerterp-Plantenga et al. (1999)^∗^	8 M 26.0 ± 2 yrs	Stay at simulated altitude		No (*ad libitum*)	5000 6000 7000 8000	d2–d6 d9–d12 d15–d19 d26–d28	–2.0 (-2.7) –2.8 (-3.8)^#^ –3.6 (-4.8)^#^ –4.7 (-6.3)^#^				
Braun et al. (1998)	16 F 21.7 ± 2.0 yrs	Stay at natural altitude		Yes (standardized diet, daily adjusted energy content to maintain body weight)	4300	12	–0.5 (-0.8) ns				
Armellini et al. (1997)	8 M, 2 F 28-49 yrs		Climbing/trekking	No	>4500	16	–3.3 (-4.7)	–1.2 (-2.2)	–2.2 (-14.7)		
Beidleman et al. (1997)	6 M 31 ± 2 yrs	Stay at natural altitude		No (free access to fluid and food)	4300	16	–1.6 (-1.9)				
Kamimori et al. (1995)	8 M 28.2 ± 3.1 yrs	Stay at natural altitude		No	4300	16	–4.9 (-5.9)		–1.2 (-8.0)		
Savourey et al. (1994)^∗^	6 M, 1 F 35.0 ± 1.7 yrs		Stay + ascents	No	4350 (3 ascents to 4807)	7	–0.7 (-1.0) ns		–0.4 (-4.1) ns		
Kayser et al. (1993)	8 M 33.7 ± 4.6 yrs	Stay at natural altitude		No (free access)	5050 5050 5050	7 14 28	–0.3 (-0.4) ns –2.7 (-3.5) –2.6 (-3.4)				
Butterfield et al. (1992)	7 M 23.7 ± 4.3 yrs (increased energy intake)		Sea level activity	Yes (prescribed and controlled food intake, increased energy intake from day 8, adjusted according to increased BMR)	4300	21 d 1- d 7 d 8- d 21	–2.1 (-2.9) –1.3 (-1.8) –0.9 (-1.2)				
Fulco et al. (1992)	16 M 27.7 (23-35) yrs		Technical climbing, hiking (4 out of 16 days)	No	3700-4300	16	–5.9 (-7.0)	–2.4 (-3.5)	–3.5 (-24.3)		
Kayser et al. (1992)	6 M 31.8 ± 4.5 yrs	Stay at natural altitude		No	5000	21	–1.9 (-2.7)				
Wolfel et al. (1991)	7 M 23 ± 2 yrs	Stay at natural altitude		Yes (controlled food intake, sufficient to cover measured energy needs)	4300	21	–2.4 (-3.3)				
Bradwell et al. (1986)	19 M, 2 F 22-56 yrs G1 (acetazolamide) G2 (placebo)		Expedition	No (unrestricted)	1300-4846 4846 (5700)	10 d ascent, 6 d stay (with 1 d to 5700 m *n* = 8) G1: G2:	(na) ↓ (na) ↓		0.2 (8.0) 0.4 (18.0)		
Fulco et al. (1985)	8 M 21.5 ± 1 (18-28 yrs)	Stay at natural altitude (relatively sedentary lifestyle)		No (*ad libitum*)	4300	18	–1.5 (-1.8)	–2.1 (-3.1)	+ 0.6 ns (+4.3)		
Stock et al. (1978)	4 M, 2 F 29-34 yrs	Stay at natural altitude		No	3650	21	–3.6 (-5.5)				
Bharadwaj and Malhotra (1974)	65 M 18-30 yrs		Ascent/stay	Yes (increased caloric intake, 4600 kcal/d)	227 to/stay at 3962-4115	8/28	–0.6 (-1.1)	–1.3 (-2.5)	–0.44 (-6.8)		–1.1 (-2.8)
Consolazio et al. (1972)	11 M 19-25 yrs G1: *N* = 5 (normal nutrient distribution) G2: *N* = 6 (G2: high carbohydrate diet)	Stay at natural altitude		Yes (3740 kcal/d)	4300	6; G1: 6; G2:	–0.9 (n.a.) –1.1 (n.a.)				
Jung et al. (1971)	8 M G1: 4 M, 24.3 ± 9.2 yrs (age < 40 yrs) G2: 4 M, 55.8 ± 6.8 yrs (age > 40 yrs)	Ascent		No	3800	4; G1: 4, G2:	–0.01 (-0,8) ns +0.05 (+3.8) ns				
Dill et al. (1969)	12 M 18-77 yrs		Scheduled exercise tests, all-out climbs, varying voluntary activity	No	3800	14	–1.9 (-2.6) ns				
Surks et al. (1966)	5 M 19-23 yrs	Stay at natural altitude		Yes (prescribed diet, ∼2999 kcal/d)	4302	8	–2.5 (-3.5)	–0.7 (-1.2) ns	–1.7 (-10.4)		–0.5 (-1.2) ns

*^∗^Study displayed in high and extreme altitude table; Studies presented in square brackets are not included in the meta-analysis due to missing data. M, male; F, female; yrs, years (mean ± SD or min-max) d, days; ns, non-significant; na, no data available; G, groups (only relevant groups from the individual studies are shown); Δkg, mean changes in BW, FFM, FM, MM and TBW before and after data collection or mean changes for defined period; Δ%, mean change in percent compared to baseline or to a defined period; BW, bodyweight; FFM, fat free mass; FM, fat mass; MM, muscle mass; TBW, total body water; G, study groups. # data used for meta-analysis for extreme altitude only. If no data were available but direction of change was illustrated, arrows are indicated: ↔ no change, ↓ decrease; BMR basal metabolic rate.*

**Table 1C:** Changes in body weight and body composition during extreme altitude.

Authors	Participants M/F, Age [years]	Type of exposure		Diet manipulation	Altitude level [m]	Duration [days]	Body weight [Δkg (Δ% of BW)]	Body composition [Δkg (Δ%)]			
		Passive	Active	Yes/No				FFM	FM	MM	TBW
**Extreme altitude**											
Levett et al. (2012)^∗^	G2 (climber) 11 M, 1 F 38.0 ± 4.9 yrs		Ascents	No	up to 6400-8848	66	–9.6 (-11.7)				
Mizuno et al. (2008)^∗^	15 M 25-45 yrs G2: *N* = 8		Group 2: climbing/ ascents	No	5250 up to 6400-7500^#^	75	–5.4 (-7.1)				
Benso et al. (2007)	9 M 40.2 ± 1.4 yrs		Expedition, (step by step acclimatization program)	No	5 M:5200-8852 3 M:5200-8600 1 M:5200-7500	49	–5.0 (-7.0)				
Reynolds et al. (1999)	6 M, 1 F 37 ± 6 yrs		Expedition	No	5300-8848	63	–7.5 (-9.5)		–4 (-39.6)	1.3 (4.0)	
Westerterp-Plantenga et al. (1999)^∗^	8 M 26.0 ± 2 yrs	Stay at simulated altitude		No (*ad libitum*)	6000 7000 8000	d9-d12 d15-d19 d26-d28	–2.8 (-3.8) –3.6 (-4.8) –4.7 (-6.3)				
											
Pulfrey and Jones (1996)	5 M, 1 F 27 ± 6 yrs		Shisha Pangma expedition	No	5900 to 8046	7	– 3.7 (na)	– 1.9 (na)	– 0.9 (na)		
Zamboni et al. (1996)	10 M, 2 W G1:5 M, 1 F (LOV) 41.2 ± 10.4 yrs G2: 5 M, 1 F (control) 35.7 ± 8,9 yrs		Expedition, ascents	Yes (G1:lacto-fish-ovo-vegetarian diet (LOV), G2: *ad libitum*)	4500-7546 ∼15 d > 5500	16; G1: 16; G2:	–3.8 (-5.2) –3.2 (-4.8)	–0.8 (-1.4) ns –1.4 (-2.6) ns	–3.1 (-19.3) ns –1.8 (-13.8) ns		
Savourey et al. (1994)^∗^	6 M, 1 F 35.0 ± 1.7 yrs		Expedition		5200 up to 8400	21	–1.5 (-2.2) ns	+0.2 (+0.02) ns			
Westerterp et al. (1994)	6 M, 4 F 35 ± 6 yrs	Stay at natural altitude (low activity level: melting snow, reading, fixing tents)		No	Stay at 6542 (5 d at 3600, 13 d ascent from 3600 to 6542)	21	–4.9 (-2.0)	–1.3 (na) ns	–3.5 (-25.7)		
Westerterp et al. (1992)	3 M, 2 F 31-42 yrs		Expedition	No	5300-8872	8	–2.2 (-4.0)	–0.8 (na)	+1.4 (na)		
Hoppeler et al. (1990)	14 M 34.9 ± 7.1 yrs		Expedition	No	stay 5200-5350 ascents up to 8000	56-70 d	–2.9 (-4.0)				
Rose et al. (1988)	6 M 28 ± 2 yrs	stay (Simulated altitude)		No (*ad libitum* diet)	0 to 8848	40	–7.4 (-8.9)	–5.0 (-7.2)	–2.5 (-17.6)		

*^∗^study displayed in high and extreme altitude table; studies presented in square brackets are not included in the meta-analysis due to missing data. M, male; F, female; yrs, years (mean ± SD or min-max); d, days; ns, non-significant; na, no data available; G, groups (only relevant groups from the individual studies are shown); Δkg, mean changes in BW, FFM, FM, MM and TBW before and after data collection or mean changes for a defined period; Δ%, mean change in percent compared to baseline or to defined period; BW, bodyweight; FFM, fat free mass; FM, fat mass; MM, muscle mass; TBW, total body water; G, study groups; BMR basal metabolic rate.*

## Risk of Bias

As the studies dealing with altitude exposure were predominantly performed in the course of mountaineering expeditions, control groups were mostly lacking, and nutritional and physical activity habits were not thoroughly controlled. Therefore, the current study largely reviews outcomes from uncontrolled before-and-after studies with a number of conditions that potentially influence body weight and composition. Thus, other sources of bias could contribute to the absence of blinding of participants and personnel or sample selection (over-representation of trained subjects). These obvious limitations have to be kept in mind during interpretation of the results.

## Meta-Analysis

Initially, a meta-analysis was performed on data from studies at all altitude levels to evaluate pooled mean changes for each outcome: bodyweight (BW), fat free mass (FFM), fat mass (FM) and muscle mass (MM). In addition, studies were stratified into three main altitude categories according to the main research question (i.e., moderate, high or extreme altitude). Within each category, pooled mean changes in BW, FFM and FM were computed. Furthermore, subgroup meta-analysis was performed for duration of stay, diet, and for active or passive exposure.

If the mean changes in BW, FFM, FM and MM were not reported in the included studies, the value of changes was calculated from the available reported values of before and after BW, FFM, FM and MM. Where standard deviation was quoted instead of standard error, the standard error was recalculated by dividing the standard deviation by the square root of the number of participants in the study.

In each meta-analysis, heterogeneity was quantified by estimation of *I*-squared (*I*^2^) as an indicator of the percentage of variation due to between-study heterogeneity that is not attributed to sampling error (Higgins et al., 2003). Random- and fixed-effects models [DerSimonian and Laird method for random-effects (DerSimonian and Laird, 1986)] were used to calculate pooled mean changes with 95% confidence intervals (CIs). If the 95% CIs did not include null, the effect estimates of the meta-analysis were considered significant. In the case of a moderate or high value of *I*^2^, the first univariate and then multivariate meta-regression (with selected significant variables from the univariate regressions; *p* < 0.25) were conducted to identify sources of heterogeneity in the effect estimates across studies (Thompson and Higgins, 2002). The following covariates were selected a priori to be included in the meta-regression analysis: baseline bodyweight (kg), altitude (m), duration (days), mean age of the subjects (years), sex, diet (manipulation/non-manipulation of diet/hypocaloric diet) and type of exposure (active/passive). Multivariate meta-regression used a residual (restricted) maximum likelihood for the measurement of between-study variance explained with Knapp-Hartung modification (Higgins and Thompson, 2004). In order to detect a publication bias, Begg’s funnel plots and the regression test of Egger et al. (1997) were applied.

All the analysis were performed using STATA (StataCorp. 2017. Release 15. College Station, TX, United States: StataCorp LLC).

## Results

The outcomes of the studies are summarized in Tables 1A–C according to the altitude categories: moderate (*n* = 13, 1500–3500 m), high (*n* = 41, 3500–5300 m) and extreme (*n* = 12, >5300 m). In addition to AL, Tables 1A–C include information on the duration of stay, type and level of physical activity, type of hypoxia and any nutritional intervention. The characteristics of the different altitude stays are outlined below:

### Moderate Altitude

Eleven of the 13 studies (Table 1A) involved physical activity. Active exposure primarily consisted of hiking, trekking and sometimes swimming (e.g., activity during moderate altitude vacations). One of the studies, which incorporated heavy exercise, also manipulated food intake (i.e., US military field ration of 17.66 MJ per day in combination with maltodextrin beverage of 5.02 MJ per day) (Hoyt et al., 1994). The duration of altitude (or simulated altitude) exposure varied from 5 to 112 days (Table 1A). The mean age of the subjects ranged from 23 to 66 years and 87% of the study subjects were male. The number of subjects varied from 5 to 35. BW was reported in twelve studies, FFM in six studies, FM in nine studies and MM in two studies.

In the studies that incorporated physical activity, there was a change in body weight of between -4.2 and +0.1 kg over an exposure time (ET) of 5 to 112 days. The two studies that investigated resting conditions (Lippl et al., 2010; Mekjavic et al., 2016) showed a weight change of -1.5 to -0.9 kg (ET: 7 to 10 days). Manipulating food intake resulted in a weight change of between -1.0 and -0.6 kg, or up to -1.8 kg when combined with heavy military exercise (ET: 5 to 21 days) (Greenleaf et al., 1978; Schena et al., 1992; Hoyt et al., 1994). One study found no reduction in body weight (Chen et al., 2010; Chia et al., 2013). Reductions in FFM were reported to range from -3.1 to -0.2 kg whereas increases in FFM ranged from 0.7 to 1.6 kg (Schena et al., 1992; Gunga et al., 2003; Chia et al., 2013). FM was investigated in nine studies where reported losses ranged from -2.6 to -0.1 kg. The MM in the one study that involved passive exposure remained unchanged (ET: 7 days) (Lippl et al., 2010). A significant reduction in MM of -1.5 kg was observed in male participants and an increase of 0.6 kg in female participants that had a prolonged active exposure to cold climate conditions (ET: 112 days) (Ocobock, 2017).

### High Altitude

Physical activity was performed during 27 of the high altitude studies, whereas 14 studies employed passive hypoxic exposures only (Table 1B). In three studies, the effects of both passive and active hypoxia were investigated (Bharadwaj and Malhotra, 1974; Debevec et al., 2014a,b). Active exposures consisted primarily of trekking or mountaineering activity with only occasional cardiovascular-based endurance exercise of low- to moderate-intensity. Fifteen studies manipulated food intake (Table 1B), two of which applied a hypocaloric diet (Fulco et al., 2002; Berryman et al., 2017). The duration of altitude (or simulated altitude) exposure varied from 4 days to 16 weeks (Table 1B). The mean age of subjects ranged from 21 to 56 years and 82% were male. The number of subjects ranged from 4 to 65. Forty-one studies investigated changes in BW, 14 in FFM, 14 in FM and one study showed changes in MM.

Body weight reductions were recorded in 38 of the 41 studies, with individual weight loss ranging from -7.7 to -0.01 kg over an ET of 4 to 75 days. Loss in body weight was more pronounced in studies that included active exposure (-7.7 to -0.01 kg) as compared to passive stays (-4.9 to -0.4 kg). A significant decrease in body weight was observed in 14 of the 15 studies with dietary manipulation. Body weight reduction ranged from -7.7 to -0.01 kg in studies where energy intake was not manipulated, and from -7.2 to -6.6 kg where a hypocaloric diet was applied (Fulco et al., 2002; Berryman et al., 2017). Body weight decreased by -3.1 to -0.4 kg in studies where dietary supplements were given (Schena et al., 1992; Vats et al., 2007; Wing-Gaia et al., 2014) as well as studies with matched food intake (Holm et al., 2010; Debevec et al., 2014a,b) and increased energy intake (Surks et al., 1966; Consolazio et al., 1972; Bharadwaj and Malhotra, 1974; Butterfield et al., 1992; Mawson et al., 2000; Barnholt et al., 2006). Of the 14 studies evaluating FFM, 13 reported a decrease (-4.6 to -0.1 kg), and one recorded a slight increase of 0.5 kg. Reductions in FFM varied between -3.9 to -0.2 kg and -2.1 to -0.1 kg in studies with active and passive exposures, respectively. FFM decreased by -2.4 to -0.4 kg when dietary intake was not manipulated (*N* = 5; including changes from control groups of dietary manipulated studies), and by -2.1 to -0.1 kg when intake was manipulated (*N* = 7) with the largest decreases (-4.6 to -3.3 kg) recorded in studies involving a hypocaloric diet (Fulco et al., 2002; Berryman et al., 2017).

Reductions in FM were reported in 12 of the 14 studies with -3.9 to -0.2 kg in studies were exercise was performed and -1.7 to -0.2 kg in studies with passive exposure. FM changed by -3.5 to +0.6 kg in studies where nutritional intake was not manipulated, and by -1.7 to 0.5 kg in studies with nutritional manitpulation.

### Extreme Altitude

Physical activity was an inherent part of most of the extreme altitude studies (9 out of 12) (Table 1C). Three studies comprised passive exposures (Rose et al., 1988; Westerterp et al., 1994; Westerterp-Plantenga et al., 1999), two were performed in a hypobaric chamber (Rose et al., 1988; Westerterp-Plantenga et al., 1999) and one included a passive stay at real altitude (Westerterp et al., 1994). Active exposure consisted primarily of trekking, mountaineering and climbing. Energy intake was manipulated in one study that provided a lacto-fish-ovo-vegetarian diet (Zamboni et al., 1996). The mean age of the subjects ranged from 23 to 41 years. The number of subjects ranged from 5 to 14, and 88% were male. The duration of altitude (or simulated altitude) exposure varied from 7 days to 10 weeks (Table 1C).

Body weight decreases were reported in all 12 studies with individual reductions between -9.6 kg and -1.5 kg. Reductions in body weight were higher in the active studies (-9.6 to -1.5 kg) when compared to studies with passive exposures(-7.4 to -2.7 kg).

Individual FFM varied by -1.9 to +0.2 kg. A decrease of -5.0 kg was reported in one study with passive hypobaric exposure at an altitude of up to 8848 m for 40 days (Rose et al., 1988). Individual FM losses ranged from -4.0 to -0.9 kg and from -3.5 to -2.5 kg in studies of active and passive exposure, respectively. MM was evaluated in one study and was shown to increase by 1.3 kg (ET: 63 days) (Reynolds et al., 1999).

## Meta-Analysis

**FIGURE 3:**
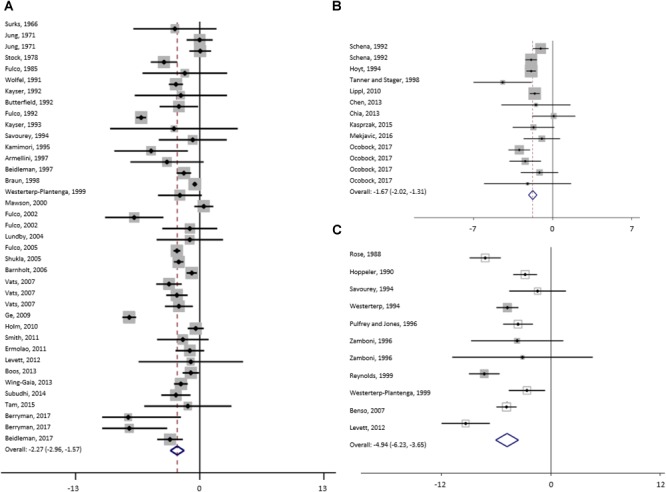
Forest plot from random-effects meta-analysis of mean bodyweight changes (95% CI) in the subgroup high **(A)**, moderate **(B)** and extreme **(C)** altitude.

**FIGURE 4:**
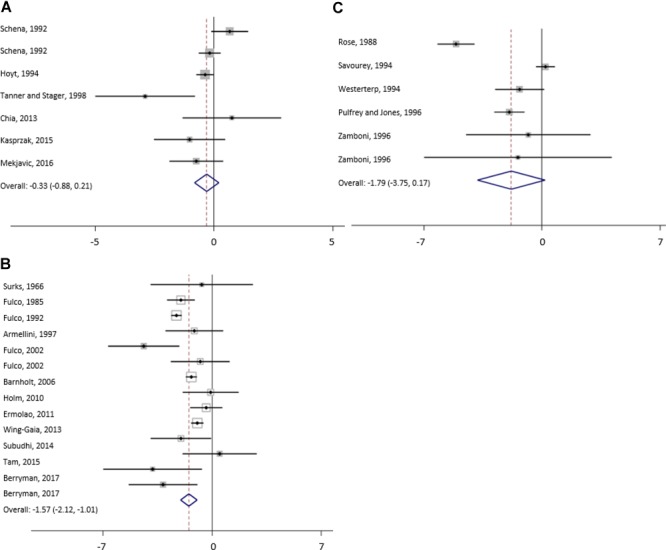
Forest plot from random-effects meta-analysis of mean fat free mass changes (95% CI) in the subgroup moderate **(A)**, high **(B)** and extreme **(C)** altitude.

**FIGURE 5:**
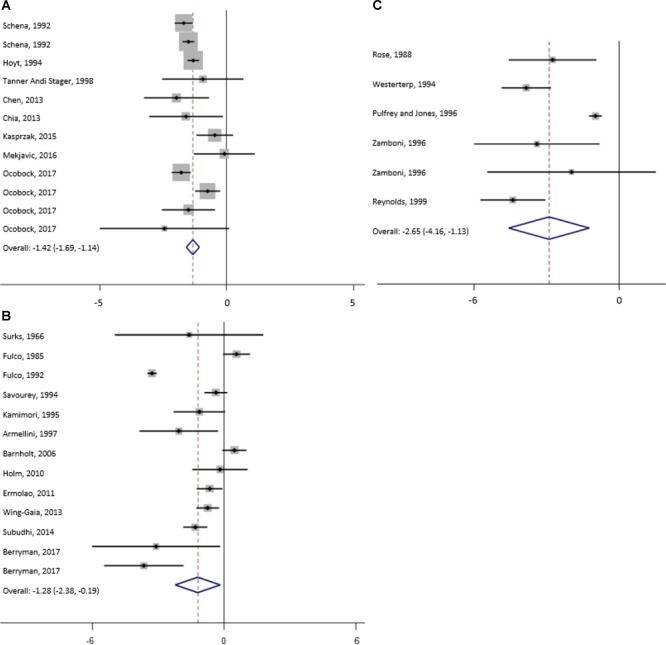
Forest plot from random-effects meta-analysis of mean fat mass changes (95% CI) in the subgroup moderate **(A)**, high **(B)** and extreme **(C)** altitude.

The results of meta-analysis of all studies and for the subgroups: moderate, high and extreme altitudes, including mean changes in BW, FFM, FM, and MM are presented in Table 2. In addition, results are shown as forest plots in Figure 3–5. A pooled mean reduction in BW, FFM, FM, and MM was observed when all studies were analyzed together (Table 2) and in each subgroup (Table 2 and Figure 3–5). A random effects model was considered as the main method for meta-analysis and meta-regression in order to account for the heterogeneity between studies. Meta-analysis of MM for the subgroup analysis was not possible due to an insufficient number of studies (only one study shown in two subgroups). Similarly, multivariate meta-regression analysis was not possible for FFM and FM for studies performed at high altitude and extreme altitude, respectively. In addition, the low number of observations in studies at high and extreme altitude did not allow univariate and multivariate meta-regression-analysis for FFM and MM. Subgroup analysis for the variables: diet, duration and type of exposure (active/passive) are presented in Table 3.

**Table 2:** Random-effects meta-analysis results.

Outcome	PMC [kg]	95% CI	*N*	*I*^2^
**BW**				
Overall	–2.6	(-3.1, -2.1)	61	96%
Moderate	–1.7	(-2.0, -1.3)	13	43%
High	–2.3	(-3.0, -1.6)	38	97%
Extreme	–4.9	(-6.2, -3.7)	11	79%
**FFM**				
Overall	–1.3	(-1.8, -0.8)	27	93%
Moderate	–0.33	(-0.9, 0.2)	7	59%
High	–1.5	(-1.0, -5.5)	14	75%
Extreme	–1.8	(-3.8, +17)	6	93%
**FM**				
Overall	–1.5	(-2.0, -1.1)	31	94%
Moderate	–1.4	(-1.7, -1.1)	12	60%
High	–1.3	(-2.4, -0.2)	13	97%
Extreme	–2.7	(-4.2, -1.1)	6	91%
**MM**				
Overall	–0.3	(-0.9, +0.4)	6	76%

*PMC, pooled mean change (95% confidence interval); *N*, number of observations; overall, meta-analysis results including all studies; BW, body weight; FFM, fat free mass; FM, fat mass; MM, muscle mass; moderate altitude (1500-3500 m), high altitude (3500-5300 m), extreme altitude (>5300 m); *I*^2^, I-squared percentage of variation across studies due to heterogeneity; No results were observed for MM in subgroups moderate, high and extreme altitude due to insufficient number of observations.*

**Table 3:** Subgroup analyses for exposure, diet, exposure and duration.

	BW	*N*	FFM	*N*	FM	*N*
	**PMC (95% CI)**		**PMC (95% CI)**		**PMC (95% CI)**	
**Moderate altitude**						
**Exposure**						
Active	–1.75 (-2.19, -1.30)	11	–0.28 (-0.89, 0.33)	6	–1.47 (-1.73, -1.21)	11
Passive	–1.46 (-1.86, -1.05)	2	–0.80 (-2.01, 0.41)	1	–0.10 (-1.37, 1.17)	1
**Diet**						
Manipulated	–1.45 (-2.23, -0.67)	2	0.09 (-0.98, 1.16)	2	–1.57 (-1.96, -1.18)	2
Non-manipulated	–1.76 (-2.21, -1.30)	11	–0.71 (-1.58, 0.16)	5	–1.32 (-1.72, -0.91)	10
Hypocaloric	n.a.		n.a.		n.a.	
**Duration [days]^∗^**						
0-7	–1.66 (1.95, -1.36)	2				
8-14	–1.20 (-2.36, -0.05)	2				
15-21	–1.48 (-2.44, -0.50)	4				
22-42	–1.40 (-4.31, 1.51)	1				
>42	–2.30 (-3.04, -1.56)	4				
**High altitude**						
**Exposure**						
Active	–2.48 (-3.47, -1.48)	25	–1.71 (-2.52, -0.90)	10	–1.88 (-3.13, -0.63)	8
Passive	–1.70 (-2.38, -1.01)	13	–1.45 (-2.04, -0.86)	4	0.05 (-0.62, 0.73)	5
**Diet**						
Manipulated	–1.40 (-2.06, -0.74)	12	–1.19 (-1.46, -0.92)	5	–0.25 (-1.15, 0.65)	4
Non-manipulated	–2.36 (-3.46, -1.26)	23	–1.51 (-2.39, -0.64)	6	–1.24 (-2.70, 0.22)	7
hypocaloric	–6.85 (-8.99, -4.72)	3	–3.94 (-5.43, -2.46)	3	–3.73 (-5.37, -2.10)	2
**Duration [days]^∗^**						
0-7	–0.37 (-1.10, 0.36)	5				
8-14	–1.62 (-2.30, -0.92)	13				
15-21	–2.68 (-3.99, -1.37)	15				
22-42	–7.05 (-7.73, -6.37)	4				
>42	–1.00 (-4.35, 2.35)	1				
**Extreme altitude**						
**Exposure**						
Active	4.87 (-6.59, -3.16)	8	–0.86 (-2.50, 0.79)	4	–2.43 (-4.47, -0.39)	4
Passive	–5.04 (-7.39, -2.70)	3	–3.18 (-6.81, 0.45)	2	–3.25 (-4.09, -2.40)	2
**Diet**						
Manipulated	–3.80 (-8.99, 1.39)	1	–0.80 (-4.42, 2.82)	1	–3.10 (-5.46, -0.74)	1
Non-manipulated	–4.99 (-6.32, -3.65)	10	–1.93 (-4.06, 0.21)	5	–2.57 (-4.23, -0.91)	5
hypocaloric	n.a.		n.a.		n.a.	
**duration [days]^∗^**						
0-7	–3.70 (-5.38, -2.02)	1				
8-14	–2.70 (-4.75, -0.66)	1				
15-21	–3.92 (-5.70, -2.14)	4				
22-42	–7.40 (-9.16, -5.64)	1				
>42	–6.05 (-8.49, -3.61)	4				

*PMC, pooled mean change (95% confidence interval); *N*, number of observations; BW, body weight; FFM, fat free mass; FM, fat mass; moderate altitude (1500-3500 m), high altitude (3500-5300 m), extreme altitude (>5300 m); n.a., no data available; ^∗^insufficient number of observations for FFM and FM.*

For studies conducted at moderate altitude, our meta-analysis reveals a statistically significant pooled mean reduction in BW of 1.7 kg (95% CI -2.0 to -1.3). A pooled mean change in FFM indicates a reduction of 0.33 kg (95% CI -0.9 to -0.21), and FM significantly decreases by, on average, -0.33 kg (95% CI -0.9 to 0.21). The largest reductions in BW were observed when exposure was active (-1.75 kg, 95% CI -2.19 to -1.30), diet not manipulated (-1.76 kg, 95% CI -2.21 to -1.30) and when duration was more than 42 days (-2.30 kg, 95% CI -3.04 to -1.56).

Results of the meta-analysis of studies at high altitude yields a significant pooled mean change in BW (-2.3 kg, 95% CI -3.0 to -1.6), FFM (-1.5 kg, 95% CI -1.0 to -5.5) and FM (-1.3 kg, 95% CI -2.4 to -0.2). Pooled mean changes in BW are most pronounced in studies that involved active exposures (-2.48 kg, 95% CI -3.47 to -1.48), when diet was not manipulated (-2.36 kg, 95% CI -3.46 to -1.26) or hypocaloric (-6.85 kg, 95% CI -8.99 to -4.72) and when duration of the exposure ranged between 22 to 42 days (-7.05 kg, 95% CI -7.73 to -6.37).

For studies conducted at extreme altitudes, there is a significant pooled mean change for BW (-4.9 kg, 95% CI -6.2 to -3.7), FFM (-1.8 kg, 95% CI -3.8 to +1.7) and FM (-2.7 kg, 95% CI -4.2 to -1.1). Reductions in BW are similar in active (-4.87 kg, 95% CI -6.59 to -3.16) and passive exposures (-5.04 kg, 95% CI -7.39 to -2.70), when diet was not manipulated (-4.99 kg, 95% CI -6.32 to -3.65) and when duration lasted between 22 and 42 days (-7.40 kg, 95% CI -9.16 to -5.64).

## Heterogeneity

Results of the analyses show a substantial and significant heterogeneity (*I*^2^) between studies, apropos BW, FFM, FM and MM at all altitude levels, and in subgroup analysis for moderate, high and extreme altitudes, with changes in BW, FFM, and FM ranging from 43 to 97% (*p* ≤ 0.05).

The effects of available covariates on between-study heterogeneity for the outcomes “BW changes” and “body compositional changes” are first derived from univariate meta-regression. Thereafter, those covariates with a statistical significance level of *p* < 0.25 are included in the multivariate meta-regression analysis for BW, FFM, and FM. Meta-regression results across all altitude levels are presented in Table 4. Results for changes in BW, FFM, and FM for studies at high- and extreme altitude subgroups are presented in Table 5.

**Table 4:** Explained between study heterogeneity (EH) by selected variables from univariate and multivariate meta-regression across all altitude levels.

Model	*N*	PMC (in kg with 95% CI)	EH	*p* value	Publication bias (Egger- Test)
**BW**					≤0.001
**Univariate**					
Manipulation of diet/	62	1.32 (0.17, 2.47)	20%	0.03	
hypocaloric diet		–4.10 (-7.50, -1.44)		0.01	
Altitude (per 1000 m)	62	–0.91 (-1.31, -0.51)	40%	≤0.001	
High altitude vs. moderate altitude	62	–0.56 (-1.73, -0.61)	30%	0.35	
Extreme altitude vs. moderate altitude		–3.26 (-4.82, -1.70)		≤0.001	
Duration (days)	62	–0.03 (-.458, -0.002)	8%	0.03	
Baseline bodyweight	57	–0.07 (-0.12, 0.01)	2%	0.06	
Sex (male)		–0.02 (-0.04, -0.002)	8%	0.03	
**Multivariate**	62		49%		
Manipulation of diet/		0.62 (-0.39, 1.64)		0.22	
Hypocaloric diet		–4.12 (-6.98, -1.27)		≤0.01	
High altitude vs. moderate altitude		–1.05 (-2.20, -0.10)		0.07	
Extreme altitude vs. moderate altitude		–3.06 (-4.46, -1.66)		≤0.001	
Duration (days)		–0.02 (-0.04, -0.002)		0.03	
Sex (male)		–0.02 (-0.03, -0.002)		0.02	
**FFM**					0.73
**Univariate**					
Manipulation of diet/	27	0.84 (-0.37, 2.04)	23%	0.16	
hypocaloric diet		–2.56 (-4.75, -0.38)		0.02	
High altitude vs. moderate altitude	27	–1.10 (-2.46,-0.21)	13%	0.10	
Extreme altitude vs. moderate altitude		–1.35 (-3.00, -0.29)		0.10	
Duration (days)	27	–0.09 (-0.17, -0.01)	17%	0.03	
Baseline bodyweight	22	–0.12 (-0.18, -0.07	71%	≤0.001	
Sex (male)	27	–0.02 (-0.04, -0.0002)	14%	0.05	
**Multivariate**	27		45%		
Manipulation of diet/		0.89 (-0.25, 2.02)		0.12	
hypocaloric diet		–1.81 (-4.02, 0.41)		0.10	
High altitude vs. moderate altitude		–1.13 (-2.32, 0.06)		0.06	
Extreme altitude vs. moderate altitude		–1.22 (-2.65, 0.21)		0.09	
Sex (male)		–0.02 (-0.04, -0.003)		0.03	
**FM**					0.48
**Univariate**					
Manipulation of diet	31	0.52 (-0.56, 1.60)	8%	0.34	
hypocaloric diet		–2.14 (-4.55, 0.26		0.08	
Altitude (per 1000 m)	31	–0.27 (-0.64, 0.11)	2%	0.16	
High altitude vs. moderate altitude	31	0.21 (-0.75, 1.18)	12%	0.65	
Extreme altitude vs. moderate altitude		–1.22 (-2.50, -0.04)		0.06	
Duration (days)			42%		
**Multivariate**	31		8%		
High altitude vs. moderate altitude		0.21 (-0.78, 1.19)		0.67	
Extreme altitude vs. moderate altitude		–1.23 (-2.52, 0.07)		0.06	
Sex (male)		0.002 (-2.98, -0.11)		0.79	

*Univariate, univariate meta-regression results; multivariate, multivariate meta-regression results; *N*, number of studies; PMC, pooled mean change; EH, explained heterogeneity in percentage; BW, body weight; FFM, fat free mass; FM, fat mass; MM, muscle mass; Manipulation of diet/hypocaloric diet was analyzed as one single covariate and compared to non-manipulation of diet; high and extreme altitude are analyzed as one single covariate and compared to moderate altitude; exposure, compared to passive exposure.*

**Table 5:** Explained between study heterogeneity (EH) by selected variables from univariate and multivariate meta-regression for high and extreme altitudes.

Model	*N*	PMC in BW	EH	*p* value	*N*	PMC in FFM	EH	*p value*	*N*	PMD in FM	EH	*p value*
		(in kg with 95% CI)				(in kg with 95% CI)				(in kg with 95% CI)		
**High altitude Univariate**												
Manipulation of diet/ hypocaloric diet	38	1.00 (-0.27, 2.27)	21%	0.12	14	0.60 (-0.53, 1.73)	42%	0.26	13	0.91 (-0.91, 2.74)	25%	0.29
		–4.47 (-7.50, -1.44)		<0.01		–2.32 (-4.32, -0.33)		0.03		–2.45 (-5.31, -0.41)		0.09
Duration (days)	38	–0.08 (-0.16, -0.12)	20%	0.02				n.s.				n.s.
Baseline bodyweight	37	–0.08 (-0.17, -0.02)	0.4%	0.13				n.s.				n.s.
Sex (male)	38	–0.02 (-0.042, -0.003)	13%	0.02	13	–0.02 (-0.03, -0.003)	66%	0.02				n.s.
Exposure (active)				n.s.				n.s.	13	–1.65 (-3.27, -0.04)	30%	0.05
**Multivariate**	38		35%						13		42%	
Manipulation of diet/		0.94 (-0.25, 2.12)		0.12		*Insufficient no. of observations*						
hypocaloric diet		–4.01 (-6.96, -1.06)		<0.01								
Duration (days)		–0.07 (-0.14,.004)		0.05								
Sex (male)										–0.02 (-0.05,.006)		0.11
Exposure (active)										–2.19 (-3.86, -0.53)		0.04
**Extreme altitude Univariate**												
Duration (days)	11	–0.05 (-0.12, -0.02)	14%	0.12				n.s.	6	–0.04 (-0.11, 0.03)	42%	0.14
Baseline bodyweight	8	–0.53 (-0.86, -0.21)	84%	<0.05				n.s.				n.s.
Age (years)				n.s.				n.s.		–0.22 (-0.41, -0.02)	77%	0.04
**Multivariate**	8		84%			*Insufficient no. of observations*				*Insufficient no. of observations*		
Duration (days)		–0.01 (-0.09, 0.07)		0.65								
Baseline bodyweight		–0.43 (-0.92, -0.05)		0.07								
Altitude (per 1000 m)		–1.10 (-5.17, 2.98)		0.50								

*Univariate, univariate meta-regression results; multivariate, multivariate meta-regression results; *N*, number of observations; PMC, pooled mean change; EH, explained heterogeneity in percentage; BW, body weight; FFM, fat free mass; FM, fat mass; MM muscle mass; moderate altitude (1500-3500 m), high altitude (3500-5300 m), extreme altitude (>5300 m); n.s., non-significant results for selected variable in meta-regression analysis; Manipulation of diet/hypocaloric diet was analyzed as one single covariate and compared to non-manipulation of diet; exposure, compared to passive exposure.*

### All Studies

Univariate meta-regression results show that when compared to non-manipulation of diet, manipulation of diet and a hypocaloric diet explains 20%, altitude (per 1000 m) 40%, altitude level (high/extreme versus moderate altitude) 30%, duration of exposure 8%, baseline bodyweight 2% and sex of subjects 8% of the total heterogeneity in BW changes between studies. Analyzing the significant covariates (manipulation of diet and hypocaloric diet, altitude level, duration and sex) in a multivariate meta-regression, the model explains 49% of the between study variance in the changes in BW.

For FFM, covariate manipulation of diet and hypocaloric diet explains 23% when compared to non-manipulation of diet, altitude level (high/extreme versus moderate altitude) 13%, duration of exposure 17%, baseline bodyweight 71% and sex 14% of the total heterogeneity between studies in a univariate meta-regression. Multivariate meta-regression, including significant covariates, reveals that manipulation of diet and addition of a hypocaloric diet together with altitude level (high/extreme versus moderate altitude) and sex explains 45% of the variance in changes of FFM between studies.

For FM, univariate regression shows that manipulation of diet and hypocaloric diet explains 8% when compared to non-manipulation of diet, altitude level (high/extreme versus moderate altitude) 12% and duration 42% of the heterogeneity. According to multivariate meta-regression, the model including significant covariates (altitude level and sex) explains 8% of the variance in the changes of FM between studies.

### Moderate Altitude

None of the selected covariates explains any of the heterogeneity between studies in BW, FFM or FM changes for the subgroup “moderate altitude.”

### High Altitude

The results of the univariate meta-regression show that manipulation of diet and hypocaloric diet explains 21%, duration of the exposure 20%, sex of the subjects 13% and baseline bodyweight 0.4% of the heterogeneity in BW changes. When significant covariates are evaluated with multivariate regression, the model including manipulation of diet or hypocaloric diet and duration of exposure explains 35% of the heterogeneity in BW changes between studies.

For FFM, most of the heterogeneity can be explained by manipulation of diet or hypocaloric diet (42%) and sex of the subjects (66%) when analyzed with univariate regression. Univariate regression reveals that manipulation of diet or hypocaloric diet explains 25% and type of exposure 30% of the heterogeneity in the changes of FM between studies. Results of the multivariate analysis show that significant covariates “sex” and “type of exposure” explain 42% of the heterogeneity.

### Extreme Altitude

With respect to BW changes at extreme altitude, univariate regression shows that baseline bodyweight explains 84% of the heterogeneity whereas duration of exposure explains 14% (non-significant). Multivariate meta-regression results reveal that 84% of heterogeneity can be explained by duration of exposure, baseline bodyweight and altitude (per 1000 m). In studies reporting FM, most of the heterogeneity can be explained by the age of the participants (77%) and by the duration of the exposure (42%) when analyzed with univariate meta-regression.

### Publication Bias

**FIGURE 6:**
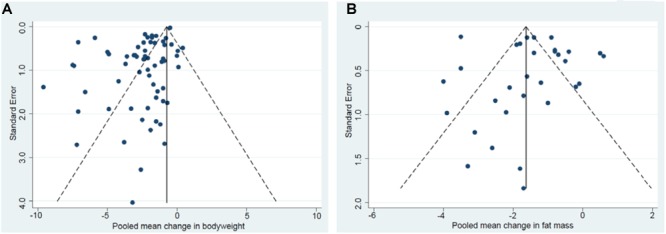
Begg’s funnel plot of changes in bodyweight **(A)**. Begg’s funnel plot of changes in fat mass **(B)**.

Beggs’s funnel plots displayed a clear asymmetry for BW (Figure 6A) and a slight asymmetry for FM (Figure 6B) indicating a lack of publications for smaller studies reporting increases in BW or FM. No significant asymmetry was found for FFM. Results from Eggers test indicate a publication bias in all studies for reports of BW changes (*p* ≤ 0.001, *N* = 62) whereas no evidence for publication bias was shown for FFM, FM and MM.

## Discussion

In summary, the results of the present meta-analysis show a decrease in pooled mean BW in the subgroups: moderate, high and extreme altitude, and where described, are accompanied by pooled mean decreases in body composition (i.e., FFM, FM), with larger changes occurring at higher altitudes. The between study heterogeneity can be explained, at least in part, by the duration of the altitude stay, the level of physical activity and nutrition.

At moderate altitude, none of the selected covariates (age, sex, baseline bodyweight, diet, active or passive exposure, duration) can explain the between study heterogeneity in BW, FFM or FM. Nonetheless, pooled mean decreases in BW are most pronounced when participants are active and when the duration of the exposure exceeds 42 days. Interestingly, at moderate altitude, clinically relevant reductions in BW of obese participants reported in the one controlled study (Greie et al., 2006) were similar to those in the normoxic control group. Only marginal reductions in FM were observed by Lippl et al. (2010) and Mekjavic et al. (2016) after 7 and 10 days of passive exposure, respectively, whereas significant reductions in FM were reported in studies that involved physical activity. Thus, the results of the studies performed at moderate altitude are not consistent in regard to the effect of hypoxia on BW or FM reduction.

At high altitude, diet (21%) and duration (20%) explains most of the heterogeneity in BW changes between studies. Sex and diet explains 66 and 42% of the between study heterogeneity in FFM changes, respectively, whereas diet and type of exposure (active/passive) explains 25 and 30% of between study heterogeneity in changes in FM, respectively. At high altitude, as at moderate altitude, the pooled mean decreases in BW were higher with active exposure and when diet was not manipulated or when the diet was hypocaloric. However, even when the caloric intake was matched or increased relative to output, BW still significantly decreased by -2.5 to -0.4 kg, indicating hypoxia to be a possible trigger for body composition changes in these studies (Surks et al., 1966; Consolazio et al., 1972; Bharadwaj and Malhotra, 1974; Wolfel et al., 1991; Butterfield et al., 1992; Barnholt et al., 2006; Holm et al., 2010). Studies that revealed the highest pooled mean reductions in BW at high altitude were those with durations of 22 to 42 days. In these studies, on average, FFM and FM affected the pooled mean body weight changes to a similar extent. There was one study of patients of type 2 diabetes mellitus that reported no changes in body weight after a 12-week expedition (De Mol et al., 2012).

At extreme altitude, a baseline BW accounts for most of the heterogeneity in BW changes between studies (84%) whereas the duration of exposure accounts for 14%. In addition, heterogeneity in FM changes between studies can be explained by the age of subjects (77%). The pooled mean decreases in BW in studies performed at extreme altitude were quite similar when physical activity was implemented or not, when diet was not manipulated and when duration lasted from 22 to 42 days. Data on MM was only reported for one study, where MM actually increased (Reynolds et al., 1999).

Reductions in BW and changes in body composition are commonly observed in high altitude research. The mechanisms leading to these changes are varied and include, but are not limited to, a higher resting metabolic rate, loss of appetite and possible impaired intestinal function at higher altitudes (Matu et al., 2018). The limitation of most of the research on this topic is that, in addition to the hypoxia level, other factors such as nutrition and exercise may greatly contribute to the observed body compositional changes. This is of particular importance as most of the studies were observational in nature, and the duration and intensity of physical activity as well as the nutritional intake were not controlled. Thus, the magnitude of their influence is difficult to assess. Nonetheless, the greatest body mass losses occurred when heavy exercise or exercise over a long duration (trekking) was incorporated and when a hypocaloric diet was applied. In contrast, the magnitude of loss was seemingly less under resting conditions and in cases where food intake was matched to energy expenditure. Interestingly, in a recent review, the degree of hypoxia was positively related to the suppression of the hunger signaling hormone acylated ghrelin (Matu et al., 2018), confirming previous findings of reduced appetite due to a reduction of the acylated ghrelin concentration following high or extreme altitude exposure over less than moderate sojourns of several days (Matu et al., 2017). This might explain the absence of significant FM reductions in the studies by Lippl et al. (2010) and Mekjavic et al. (2016) with passive exposures to a moderate altitude.

Significant losses of FFM and MM were observed at altitudes as low as ≤5000 m (Ocobock, 2017), with the extent dependent on duration, temperature and body fat percentage at the outset. These results were confirmed in a very recent study that showed that there is muscle wasting even during short simulated exposures (21 days) to an altitude of only 4000 m under strictly controlled and standardized environmental, dietary and activity conditions (Debevec et al., 2018). In the present review, only 5 studies reported data on TBW. Fluid loss equivalent to up to 1-2 kg of body weight is reported to accompany the adaptation to altitude (Kayser, 1994). Overall, maximum TBW loss was similar under moderate and high altitude conditions (approximately -0.7 L(ns) to -1.1 L), whereas no changes were reported at extreme altitudes. The fluid loss is suggested to be based on hormonally induced diuresis and natriuresis, decreased voluntary salt and water intake and increased insensible water loss (respiratory and surface water loss) (Hoyt and Honig, 1996), though exercise and/or hypertension may affect these processes (Ledderhos et al., 2002). As a consequence, plasma volume, interstitial and intracellular fluid volumes decrease, whereas hemoglobin concentration, mitochondrial- and capillary density increase, potentially resulting in increased skeletal muscle oxygen conductance and facilitated diffusion (Hoyt and Honig, 1996). Such changes might be beneficial for physical performance, however, detrimental effects due to excessive dehydration at altitude may occur.

One very important factor that one should not ignore when discussing body composition changes in lowlanders exposed to high altitudes is the general influence of genetic involvement in the adaptation to hypoxia. In high altitude natives, increased lung capacity (due to larger chests) was shown to be accompanied by increased diffusion capacity for oxygen (Greksa et al., 1994), which would imply a lesser degree of hyperventilation during hypoxia and, consequently, a lesser insensible water loss. In addition, metabolic factors such as fuel preference for carbohydrates at rest and during exercise may be involved in body composition adaptations in highlanders (Watt et al., 1976). Thus, changes in body composition may not only be based on an adaptive response to hypoxia but may also depend on different genetic factors.

### Practical and Clinical Implications

It is not entirely clear whether the duration of the altitude stay or the severity of hypoxia is the more potent trigger in initiating weight loss. In regard to moderate altitude conditions, prolonged sojourns, especially if combined with hiking activities, may reduce BW and FM. However, these changes may not be induced by hypoxia alone since comparable hiking studies under normoxic conditions showed similar results. Moderate altitudes seem not to meaningfully decrease FFM and MM or affect appetite. When going to high altitude, climbers should consider that an increased BMR in conjunction with higher energy demands due to physical activity and inadequate energy intake - possibly due to loss of appetite - can lead to a negative energy balance resulting in loss of FM and FFM. Even during passive high and extreme altitude sojourns with matched or increased energy intake, changes in body composition, including reductions in FFM and FM, may occur. This emphasizes the importance of adequate nutritional intake, especially during active altitude exposure, to keep weight loss and, thus, FFM and MM reductions, which might negatively affect mountaineering performance and safety, to a minimum. Focus has to be also put on appropriate fluid intake as reduced fluid intake and increased fluid loss may lead to BW loss due to dehydration, which again may negatively affect performance and safety. Yet potential positive effects on performance, as outlined before, might also be anticipated. In addition it has to be mentioned that due to individual adaptation to hypoxia, the effects on body composition and BW may vary greatly between individuals exposed to high and extreme altitudes.

The observations of an increased energy expenditure, increased lipid metabolism and decreased glycogen metabolism following passive hypoxic conditioning (Workman and Basset, 2012) may lead to the assumption that, if applied with an adequate duration and severity, hypoxia could induce favorable weight loss in obese or overweight persons. The underlying mechanism leading to weight reduction during altitude sojourns is not entirely clear, and the effect of hypoxia *per se* remains unexplained. Fluid loss from all compartments (extra- and intracellular) as a response to hypoxic exposure might improve oxygen delivery due to reduced diffusion distances (Hoyt and Honig, 1996) and thus might facilitate a shift in fuel metabolism towards fat. The altitude specific effect (normobaric hypoxia) for potential weight loss programs in overweight persons was recently appraised by a systematic review (Hobbins et al., 2017).

Some limitations of the review have to be acknowledged. Even though a systematic literature search was applied, there is the possibility that some relevant studies were not included in this meta-analysis if our search algorithm did not capture them. A significant publication bias was detected in all studies for BW changes. Additionally, and as already mentioned in the introduction section, it is not possible to unequivocally identify the influence of AL alone on body compositional changes due to the nature of most of the included studies (i.e., uncontrolled observational studies). Nevertheless, this analytical review gives a synopsis of the body compositional changes at various altitudes under different field conditions with a focus on the role of physical activity, duration of the exposure and nutrition.

In conclusion, BW and body composition changes occur during sojourns at different altitudes. The mechanisms leading to these changes are manifold and the magnitude of change is, in addition to the individual adaptive response to hypoxia, greatly influenced by the AL, the duration of the stay, the level of physical activity and the nutritional intake. This review identifies at least two contrasting motivations for hypoxia exposure; the experience of high altitude mountaineering and the “therapeutic” effect of hypoxia (e.g., weight loss programs). This analytical review of published data highlights the notion that adequate nutritional intake during high altitude expeditions should be assured in order to prevent negative energy balance. Furthermore, there is currently no convincing evidence for the use of hypoxic treatments for obesity that do not involve any lifestyle modification and, as such, presents a focus for further investigations.

## Author Contributions

All authors listed have made a substantial, direct and intellectual contribution to the work, and approved it for publication.

## Conflict of Interest Statement

The authors declare that the research was conducted in the absence of any commercial or financial relationships that could be construed as a potential conflict of interest.
